# Evaluation of an automatic needle‐loading system

**DOI:** 10.1120/jacmp.v5i2.1971

**Published:** 2004-08-16

**Authors:** Janelle Morrier, Nicolas Varfalvy, Mario Chrétien, Luc Beaulieu

**Affiliations:** ^1^ Département de Radio‐Oncologie et Centre de Recherche en Cancérologie Hôtel‐Dieu de Québec 11 Côte du palais Québec Quebec Canada G1R 2J6; ^2^ Département de physique génie physique et optique Université Laval Québec Quebec Canada G1K 7P4

**Keywords:** brachytherapy, I‐125, safe seed handling, radiation safety, seed assay

## Abstract

The purpose of this paper is to evaluate the dosimetric capabilities and the radiation protection (RP) performance of a new automatic needle‐loading system for permanent prostate implants, the Isoloader (Mentor Corp.). The unit has been used in more than 100 clinical cases at our institution.

The Isoloader is a computerized workstation that allows automated seed testing by a solid‐state CdZnTe radiation detector and loading in surgical needles. The seeds are received in a shielded and ready‐to‐use cartridge. Radiation protection measurements were done on a cartridge filled with 67 I125 seeds and during dosimetric seed verification and needle loading. The reproducibility of the detector was tested and its accuracy was determined by comparison to specified activities of six calibration seeds and to their measurements in a calibrated well‐chamber (WC). Finally, the times required to complete dosimetric verification and needle loading were evaluated.

The cartridge was found to be adequately shielded, since no significant amount of radiation was detected around it. Radiation during seed assay was found to be worst at the cartridge's bottom, where it has a value of 15.2 μSv/h (1.4 μSv/h at 10 cm). For the needle‐loading task, measurements were performed with a typical needle (three seeds) at the shielded needle holder surface yielding

307.2 μSv/h (8.3 μSv/h at 20 cm). Seed dosimetric verification takes an average of 15 s/seed, while it takes a mean time of 50 s/needle to complete the loading task. Measurements of the six seed activities were within 0.65% of the ordered activities and 1.9% higher on average than those from the WC (min=0.7%;max=3.5%). The reproducibility of the measurements of the CdZnTe detector was excellent, with an average of 0.01% of deviation from a reference measurement (N=120;σ=1.9%).

We therefore conclude that the Isoloader is a safe, fast, and effective needle‐loading system.

PACS number: 87.53.Jw

## I. INTRODUCTION

Because of their proven efficiency, permanent prostate implants have recently become more and more common. The number of patients treated by this technique in 2002 at our institution was almost twice that of 1999, and this number is expected to continue to grow during the years to come. In fact, some studies show that this technique presents cancer control and survival rates for selected patients (clinically organ‐confined, Gleason grade <7, PSA <10 ng/mL) that are equivalent to those of surgery and external radiation therapy with a lower rate of complications.^(^ ^1^ ^–^ ^6^ ^)^

The technique consists of inserting several I125 seeds in the prostate under transrectal ultrasound guidance. Implantation is achieved using needles filled with seeds and inserted through the perineum. Because the preparation of these needles requires seed manipulations, the growing interest surrounding the permanent implant technique makes the safe handling of sources a major concern.

In the past few years several types of applicators have been developed, such as the Mick applicator. With the Mick applicator the seeds were implanted one by one in the prostate; however, most centers changed to different manual loading systems. Manual loading systems allowed the preloading of needles using suction (SeedVac™ from Standard Imaging Inc.) or sliding (Seed Slider™ from Standard Imaging Inc.) systems. With these systems, the seeds had to be sterilized after the user had verified their activity with a well‐chamber before they were loaded in the needles. In the last year, a third loading device system has surfaced: an automatic needle‐loading system that avoids direct handling of the seeds. In addition, this new technology not only loads needles, but also performs automated seed dosimetric verification while preserving sterile conditions. The new system can also be equipped with an ultrasound image acquisition module as well as a dosimetric planning component. Our apparatus was not equipped with these modules; therefore, they are not part of this study.

The Isoloader (Mentor Corp.) was the first of its kind to enter the market, and our institution was the first to acquire one. The Isoloader is a computerized workstation that allows automated seed testing by a solid‐state CdZnTe radiation detector and loading in surgical needles following the treatment plan while preserving sterile conditions. The seeds are received in a sterilized, shielded, and ready‐to‐use cartridge, which is individually prepared for each patient. The unit is operational as soon as the cartridge has finished its initialization procedure, which mainly consists of a data transfer between the cartridge and the apparatus as well as a mechanical check of the components. The initialization procedure generally takes less than 2 min.

The dosimetric capabilities and the radiation protection features of this new system have been tested to verify their performances; the times required to complete dosimetric verification and needle loading were also evaluated. This paper presents the results of these investigations and discusses the clinical impacts of such a system, given that it has been used for more than 100 clinical cases at our institution.

## II. METHODS

The tests performed on the unit were divided into three major sections: dosimetric, radiation protection, and time evaluation.

The dosimetric test evaluated the reliability of the solid‐state CdZnTe radiation sensor integrated in the unit. The detector is able to measure X‐ and gamma‐rays from 10 keV to 1 MeV. The allowed apparent activity range is 5.18 MBq to 19.24 MBq for I‐125 (3.7 MBq to 76.59 MBq for Pd‐103), but the Isoloader user manual specifies that there is no detectable departure from linearity over this range. The accuracy and the reproducibility of the detector were tested using six calibration sources ranging from 10.286 MBq to 23.014 MBq. Accuracy was determined by comparison of the Isoloader measurements with the specified seed activities and with measurements from a calibrated Victoreen 34‐070‐5000 well‐chamber (WC) used with an electrometer from Standard Imaging (model MAX 4000). To determine the reproducibility, 20 consecutive measurements of each seed were taken. Each of these was then compared with the first one, which was taken as the reference.

**Figure 1:**
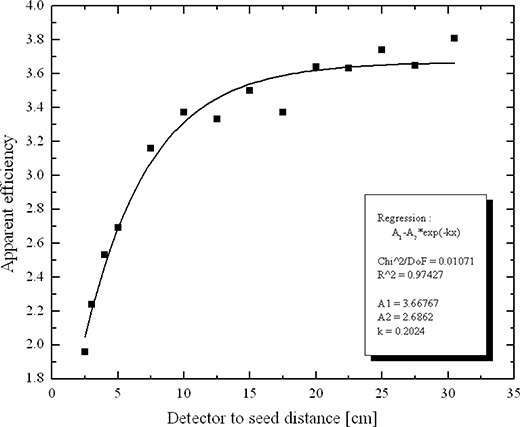
Calibration curve of the Inspector detector giving the apparent efficiency as a function of the detector to seed distance. The line represents a fit to the data.

The goal of the radiation protection (RP) evaluation was to ensure that utilization of this new system helped to significantly reduce the radiation exposure of the technologist in charge of needle preparation. Measurements using a Geiger‐Müller detector (Inspector™ from Radiation Alert) were done on a cartridge filled with 67 I‐125 seeds of 22.015 MBq (1475.01 MBq total activity). To precisely determine the efficiency of the detector for I‐125 gammas and the geometry factors to be used at short distances, a calibration curve (Fig. 1) was obtained using two seeds of known activities (6.77 MBq and 28.93 MBq). Here, apparent activity stands for a combination of intrinsic detector efficiency for I‐125 and geometry factor. Of course, at large distances the geometric factor is constant. Based on this curve, we decided to use only the asymptotic value of 3.67 (367%) because it represents an overestimation for small distances. Thus, our measurements should be seen as conservative estimates, which is appropriate in RP. Other RP evaluations were performed during seed dosimetric verification and during needle loading. For the needle‐loading task measurements were done both in a typical case of a three‐spaced seed needle and in a worst‐case scenario of a 10‐seed needle.

Finally, the times required to perform seed dosimetric verification and needle loading were evaluated to determine whether the Isoloader could be used in the operating room (OR), where time is of the utmost importance.

## III. RESULTS AND DISCUSSION

### A. Dosimetric features

The measured activities for the six calibration sources were on average within 0.625% (σ=1.32%,min=0%,max=2.46%) of the ordered activities and systematically higher than those from the WC (mean deviation=1.89%,σ=0.92%,min=0.69%,max=3.48%), which is well within the precision of the WC. The complete results are presented in Tables 1 and 2, where the Isoloader and the WC measurements are the mean values of 20 measurements. The CdZnTe detector was thus found to have a good accuracy.

**Table 1:** Deviations of Isoloader readings when compared to the specified activities of 6 calibration seeds

Seed	Specified activities (MBq)	Isoloader measurements^a^ (MBq)	Deviation (%)
1	15.503	15.577	0.47
2	7.511	7.696	2.46
3	6.623	6.660	0.59
4	6.993	7.104	1.59
5	5.920	5.920	0.00
6	5.439	5.365	–1.36
		Mean deviation:	0.625

^a^ Mean over 20 measurements.

**Table 2:** Deviations of Isoloader readings of 6 calibration seeds when compared to those from a well‐chamber

Seed	Well‐chamber measurements^a^ (MBq)	Isoloader measurements^a^ (MBq)	Deviation (%)
1	15.355	15.577	1.45
2	7.437	7.696	3.48
3	6.549	6.660	1.69
4	6.956	7.104	2.13
5	5.809	5.920	1.91
6	5.328	5.365	0.69
		Mean deviation:	1.89

^a^ Mean over 20 measurements.

**Figure 2:**
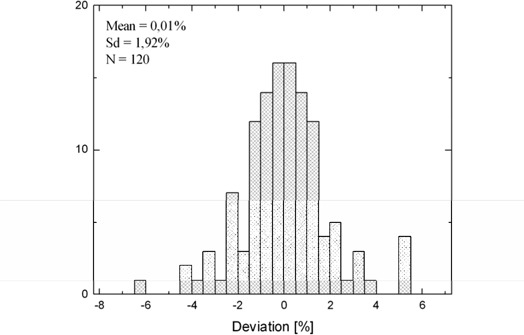
Reproducibility of the CdZnTe detector. Deviations of measurements from the first one taken as reference

Figure 2 shows a measurement deviation histogram which was done to evaluate the reproducibility. On average, the measurements were 0.01% higher than the reference measurement (N=120; σ=19%). We thus conclude that the reproducibility of the Isoloader detector is excellent.

### B. Radiation protection

When measuring around a cartridge filled with 67 seeds, no radiation above the background was observed. This means that the cartridges are adequately shielded and that there is no need for special precautions to manipulate it.

**Figure 3:**
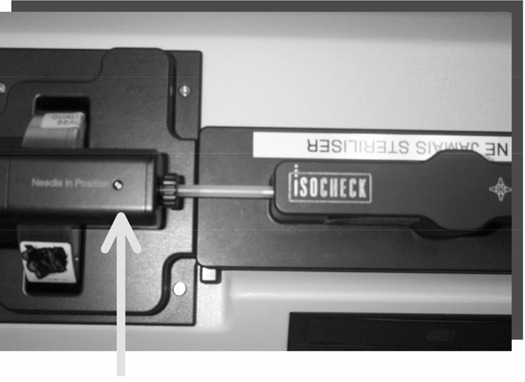
Position of maximal radiation level during seed dosimetric verification

Measurements during the seed dosimetric verification process showed a maximal radiation level of 18.2 μSv/h at the cartridge's bottom surface (Fig. 3) and 1.7 μSv/h at a distance of 10 cm from the surface. This level of radiation is small enough that a reasonable working distance is a sufficient protection.

**Figure 4:**
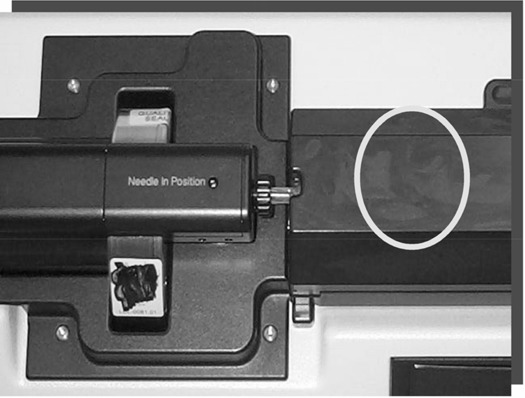
Position of maximal radiation level for the needle‐loading task

For the needle‐loading task, measurements were taken at the shielding surface (Fig. 4), where the radiation was the highest, for two different needle configurations: a typical one of three spaced seeds and another containing ten back‐to‐back seeds representing an extreme scenario. With the standard needle in place, the radiation level at the shielded needle holder surface was 368.4 μSv/h (9.99 μSv/h at 20 cm). For the extreme scenario, the radiation level at the needle holder surface was higher than 1 mSv/h (35.3 μSv/h at 20 cm).

The needle holder shielding was found to be insufficient to provide adequate protection to the radiation technologist in charge of needle preparation. Consequently, Mentor Corp. developed a new shielding. The only difference between the new shielding and the old one is the addition of a 1 mm plate of stainless steel in the cover. It was tested with the extreme scenario needle of 10 seeds, and the radiation level was significantly lower: 26.3 ?Sv/h at the surface and 0.62 ?Sv/h at 20 cm. With this modification, a reasonable working distance can be considered a sufficient protection for the needle‐loading task as for dosimetric verification.

To ensure that a reasonable distance is maintained by the technologist operating the apparatus, one restriction was imposed. The Isoloader has a touch‐screen interface, so no other applications are permitted to be run during seed assaying and/or needle loading. This is particularly important when the apparatus is equipped with the two extra modules discussed in the Introduction. With those components installed, one could be tempted to, for instance, use the planning algorithm while assaying the seeds, which would expose the operator to an unnecessary amount of radiation. With the exception of this rule, no other restrictions are needed to ensure a safe use of the apparatus.

### C. Time evaluation

Dosimetric verification of a single seed takes an average of 15 s. This makes it possible to test 100% of the seeds for each patient within a reasonable time (generally less than 15 min). In the past, only 10% of the seeds were assayed at our institution (as recommended by the AAPM task group No. 56).(7) From a dosimetric point of view, the verification of all seeds for a patient is a major advance. Needle loading takes 10 s to 60 s, depending on its seed and spacer content. To estimate the time required to complete the loading of all needles for a patient, one should calculate an average of 50 s/needle (or 18 s/seed), which is the average time required including the manipulation of the needles themselves.

### D. Clinical impact

As expected with any new development or technology, the Isoloader has had a significant impact on our clinical methods and procedures. For instance, the speed of seed dosimetric verification allowed us to begin verifying 100% of the seeds for each patient. This could eventually allow us to readjust the dosimetric plan on the basis of the new information to get a more realistic idea of the dosimetry for each patient. This possibility may thus become a new dosimetry avenue.

Furthermore, a detailed radiation report is produced after each cartridge assay. This report was a strong addition to our quality assurance program and makes a welcome supplement to the patient medical file.

One of the most important clinical impacts of the Isoloader is that the times required to carry out seed assaying and needle loading were short enough such that the Isoloader can be used in the OR (for seed assaying and needle loading). This has been the case at our institution since May 2003, representing over 50 clinical cases. Accomplishing these procedures in the OR allows us to considerably reduce the preparation prior to entering the OR, resulting in savings in technologist hours. Moreover, because the apparatus allows maintenance of sterile conditions throughout the procedure, seed sterilization is no longer necessary. With the addition of a dosimetry system to our machine, the apparatus may allow us to do a real‐time treatment planning in the future.

## IV. CONCLUSION

The results presented in this paper allow us to conclude that the Isoloader is a safe, fast, and effective needle‐loading system. The radiation protection aspect of our study showed that the radiation level at the surface of a cartridge was small enough to consider negligible. The levels due to seed assaying and needle loading were also found sufficiently small to ensure an acceptable protection. Our investigation also showed that the times required to perform dosimetric seed verification and needle loading with the Isoloader are short enough to be performed in real time in the OR and to permit a complete (100%) seed verification for each patient. Finally, our study illustrates the good accuracy and reproducibility of the CdZnTe detector.

## ACKNOWLEDGMENTS

The authors would like to thank Mentor Corp. for taking into account our findings and producing a new shielding and for providing calibration seeds. The authors would also like to recognize the work of the technologist, whose cooperation has been of great help.

Finally, special thanks to Ghyslain Leclerc for his constructive and helpful comments on the manuscript.
